# Assessing the Impact of the COVID-19 Pandemic in Spain: Large-Scale, Online, Self-Reported Population Survey

**DOI:** 10.2196/21319

**Published:** 2020-09-10

**Authors:** Nuria Oliver, Xavier Barber, Kirsten Roomp, Kristof Roomp

**Affiliations:** 1 The Institute for Human(ity)-Centric Artificial Intelligence ELLIS Unit Alicante Foundation Alicante Spain; 2 Center of Operations Research Universidad Miguel Hernández Elche Spain; 3 Luxembourg Centre for Systems Biomedicine University of Luxembourg Belvaux Luxembourg; 4 Microsoft Redmond, WA United States

**Keywords:** COVID-19, SARS-CoV-2, public health authorities, large-scale online surveys, infectious disease, outbreak, public engagement, disease prevalence, impact, survey, spain, public health, perception

## Abstract

**Background:**

Spain has been one of the countries most impacted by the COVID-19 pandemic. Since the first confirmed case was reported on January 31, 2020, there have been over 405,000 cases and 28,000 deaths in Spain. The economic and social impact is without precedent. Thus, it is important to quickly assess the situation and perception of the population. Large-scale online surveys have been shown to be an effective tool for this purpose.

**Objective:**

We aim to assess the situation and perception of the Spanish population in four key areas related to the COVID-19 pandemic: social contact behavior during confinement, personal economic impact, labor situation, and health status.

**Methods:**

We obtained a large sample using an online survey with 24 questions related to COVID-19 in the week of March 28-April 2, 2020, during the peak of the first wave of COVID-19 in Spain. The self-selection online survey method of nonprobability sampling was used to recruit 156,614 participants via social media posts that targeted the general adult population (age >18 years). Given such a large sample, the 95% CI was ±0.843 for all reported proportions.

**Results:**

Regarding social behavior during confinement, participants mainly left their homes to satisfy basic needs. We found several statistically significant differences in social behavior across genders and age groups. The population’s willingness to comply with the confinement measures is evident. From the survey answers, we identified a significant adverse economic impact of the pandemic on those working in small businesses and a negative correlation between economic damage and willingness to stay in confinement. The survey revealed that close contacts play an important role in the transmission of the disease, and 28% of the participants lacked the necessary resources to properly isolate themselves. We also identified a significant lack of testing, with only 1% of the population tested and 6% of respondents unable to be tested despite their doctor’s recommendation. We developed a generalized linear model to identify the variables that were correlated with a positive SARS-CoV-2 test result. Using this model, we estimated an average of 5% for SARS-CoV-2 prevalence in the Spanish population during the time of the study. A seroprevalence study carried out later by the Spanish Ministry of Health reported a similar level of disease prevalence (5%).

**Conclusions:**

Large-scale online population surveys, distributed via social media and online messaging platforms, can be an effective, cheap, and fast tool to assess the impact and prevalence of an infectious disease in the context of a pandemic, particularly when there is a scarcity of official data and limited testing capacity.

## Introduction

### Background

The first cases of COVID-19 were reported in Wuhan, China in December 2019. Since then, the SARS-CoV-2 virus has spread worldwide, infecting over 24 million people and causing over 825,000 deaths worldwide as of August 27, 2020 [1]. This virus has caused significantly more infections and deaths, compared with previous outbreaks of other coronaviruses causing severe acute respiratory syndrome and Middle East respiratory syndrome. The World Health Organization declared a global COVID-19 pandemic on March 11, 2020, and to date has been unable to predict the duration of the pandemic [2].

The first confirmed case of COVID-19 in Spain was reported on January 31, 2020, when a German tourist tested positive in the Spanish Canary Islands. However, this was an isolated imported case. It was not until February 24 when Spain confirmed several new COVID-19 cases related to a recent SARS-CoV-2 outbreak in the north of Italy. Since that date, the number of COVID-19 cases grew exponentially in Spain so that by March 30, 2020, there were over 85,199 confirmed cases, 16,780 recoveries, and the staggering figure of 7424 deaths, according to the official numbers. On March 25, 2020, the death toll attributed to COVID-19 in Spain surpassed that of mainland China, and it was only surpassed by the death toll in Italy. The economic and social impact of the COVID-19 pandemic in Spain is without precedent.

To combat the pandemic, the Spanish Government implemented a series of social distancing and mobility restriction measures. First, all classes at all educational levels were cancelled in the main hot spots of the disease on March 10, in the Basque Country and on March 11, 2020, in the Madrid and La Rioja regions. All direct flights from Italy to Spain were cancelled on March 10. On March 12, the Catalan Government quarantined four municipalities that were particularly affected by the virus. On March 13, the Government of Spain declared a state of emergency for 2 weeks across the entire country. Since the state of emergency was established, all schools and university classes were cancelled, large-scale events and nonessential travel were forbidden, and workers were encouraged to tele-work. Despite these efforts, the daily growth rate in the number of confirmed COVID-19 cases continued to grow. Thus, on March 30, new mobility restriction and social distancing measures were implemented; all nonessential labor activity was to be interrupted for a 2-week period. Moreover, the Spanish Government extended the state of emergency first until April 11 and then renewed on a biweekly basis until June 21. Although these interventions put a halt to the normal daily lives of most people in Spain, their impact on people’s economic, physical, and mental well-being were unknown at the time, as was the actual prevalence of the disease.

Given the growth rate in the number of confirmed COVID-19 cases, rapid assessments of the population’s situation and perceptions of the infection are of paramount importance. Traditional methods, such as population-representative household surveys are slow to design and deploy [3]. Phone surveys are generally faster to conduct, yet they are labor intensive and often yield low response rates (as low as 10% or less [4]). Moreover, the resulting sample might be biased and difficult to reweight [5]. Given the limitations of these traditional methods and given the need for rapid data collection, large-scale online surveys can be a valuable method to quickly assess and longitudinally monitor the situation and perceptions of the population in the context of a pandemic [6]. Thus, to shed light on important, yet unknown, questions related to COVID-19, we designed a 24-question online survey, called the *Covid19Impact* survey, to be targeted to the Spanish population. The survey became viral 12 hours after its publication, yielding over 140,000 answers. It is one of the largest surveys in the world carried out in the context of the COVID-19 pandemic [7].

### Population Surveys During the COVID-19 Pandemic

Other efforts to collect data from the population regarding the COVID-19 pandemic have been deployed in multiple countries. The largest study to date involved the *Methods* smartphone app, with 2,618,862 participants who self-reported symptoms in the United States and the United Kingdom [8]. The study asked questions focused on risk factors and symptoms, and described a predictive model of COVID-19 based on these variables. In Canada, *FLATTEN* [9] has gathered data from respondents and asks simple health and demographic-related questions to help monitor the spread of the virus in an anonymous manner. The *International Survey on Coronavirus* asks questions focusing on the psychological impact of the crisis [10]. There were three main findings from the analysis of this survey’s answers: many respondents found that both the population and their governments’ response to the COVID-19 pandemic was insufficient, this insufficient response was associated with lower mental well-being, and a strong government response was associated with an improvement in respondents’ views of other people and their government together with better mental well-being. The *COVID-19:CH Survey* in Switzerland aims to collect personal data related to COVID-19 testing with additional health- and potential exposure–related information [11]. The data collected is presented to the public in a visual format, giving information on, among other things, demographics, comorbidities, and symptoms. In Israel, the Weizmann Institute and the Ministry of Health are collecting data on basic demographics, health, and potential exposure [12]. The project aims to predict the location of COVID-19 outbreaks by analyzing information collected about the virus symptoms and public behavior in real time [6,13,14].

Numerous efforts with smaller numbers of respondents have also taken place or are ongoing. In China, an early study was conducted between January 27 and February 1, 2020, which relied on the Chinese social media and traditional media outlets asking about knowledge, attitudes, and practices toward COVID-19 [15]. Among its many findings, the authors reported that most respondents felt that China could win the battle against the virus. An early international project was run from February 23 to March 2, 2020, collected data from the United Kingdom and the United States using an online platform managed by Prolific Academic Ltd, and asked about knowledge and perceptions of COVID-19 [16,17]. The survey provided potential information to guide public health. In mid-March and over 48 hours, responses were collected in the United States; the survey had been posted on 3 social media platforms (Twitter, Facebook, and Nextdoor) and collected data on symptoms, concerns, and individual actions [18]. They showed that 95.7% of respondents made lifestyle changes, including handwashing, avoiding social gatherings, social distancing, etc. In the United Kingdom, data was collected attempting to identify sociodemographic adoption of social-distancing measures, ability to work from home, and both the willingness and ability to self-isolate [19], providing potential information to policy makers. An online survey (*FEEL-COVID*) used the snowball sampling method to collect data in India and found that almost one-third of respondents were negatively psychologically impacted by the pandemic [20].

Our work complements these previous related efforts by focusing on Spain (one of the most affected countries by the COVID-19 pandemic) and by addressing four areas of people’s experiences during the confinement: their social contact behavior, economic impact, labor situation, and health status.

### This Study

Despite the availability of data regarding the number of confirmed COVID-19 cases, hospitalized and intensive care patients, and deaths in the early stages of the COVID-19 pandemic, there was a scarcity of high-quality data about important questions related to the population’s experience.

First, there is the issue of underreporting confirmed cases and COVID-19–related deaths. Work by the Imperial College COVID-19 Response Team [21] estimated that 15% of the Spanish population could be infected by SARS-CoV-2. However, this figure was estimated to be much lower at around 5.3% by the preliminary results of a seroprevalence study carried out by the Spanish Ministry of Health [22,23]. Assessing the percentage of infected individuals is of utmost importance to build accurate epidemiological models and to assist policy makers in their decisions.

Second, there are unknowns regarding the sources of infection. Are people being infected by friends, family members, relatives, and coworkers, or are they being infected when shopping in supermarkets or at the bakery? The effectiveness of different government interventions will depend on the answers to these questions.

Third, the economic impact that the COVID-19 crisis will have on people’s lives is yet to be quantified. According to the latest figures from the Spanish Industry, Commerce and Tourism Ministry, only 0.2% of Spanish companies have 250 or more employees, 44.6% of companies are micro (1-9 employees) or small (10-49 employees), and 54.4% of companies consist of the self-employed [24]. Small businesses are generally unprepared to confront such a crisis. Moreover, tourism represents 14.6% of Spanish gross domestic product (GDP) and 2.8 million jobs, and these are threatened by the COVID-19 pandemic [24]. Measuring the impact that the pandemic is having on people’s finances is of great value to policy makers. Finally, there is the personal experience related to having to be confined in the home for weeks. How much longer will the population be able to sustain this situation?

In this paper, we describe the *Covid19Impact* survey, which was designed to answer these questions. We present the methodology that we followed to gather a large-scale sample via an online survey, followed by the analysis of the resulting answers and the main insights derived from them. Finally, we describe our conclusions and lines of future work.

## Methods

### Sampling and Data Collection

To answer the previously formulated questions, we designed a 24-question anonymous online survey that we refer to as the *Covid19Impact* survey (Multimedia Appendix 1). The survey is divided in 4 sections that address four different dimensions related to the population’s experience during the COVID-19 crisis: their social contact in the last 2 weeks, the economic impact of the pandemic, their workplace and labor situation, and their health status. Moreover, the survey collects basic demographic (age range, gender, postal code) and home (type of home and number and ages of people in the home) data.

We used the self-selection online survey method of nonprobability sampling to recruit participants via social network posts (mainly Twitter and WhatsApp), asking the Spanish population (18 years or older) to answer the survey. This sampling method is particularly suitable during a confinement situation where the mobility and social contact of the population is greatly reduced. Thus, the online distribution of the survey enabled fast access to it by large numbers of people.

In addition to distributing the survey on Twitter and WhatsApp, we used snowball sampling [25]. The goal was to collect as large of a sample as possible in a short amount of time, as the COVID-19 situation was rapidly evolving, and new government measures might be required. The objective is to gather a snapshot of people’s experiences regarding the four sections previously described.

**Figure 1:**
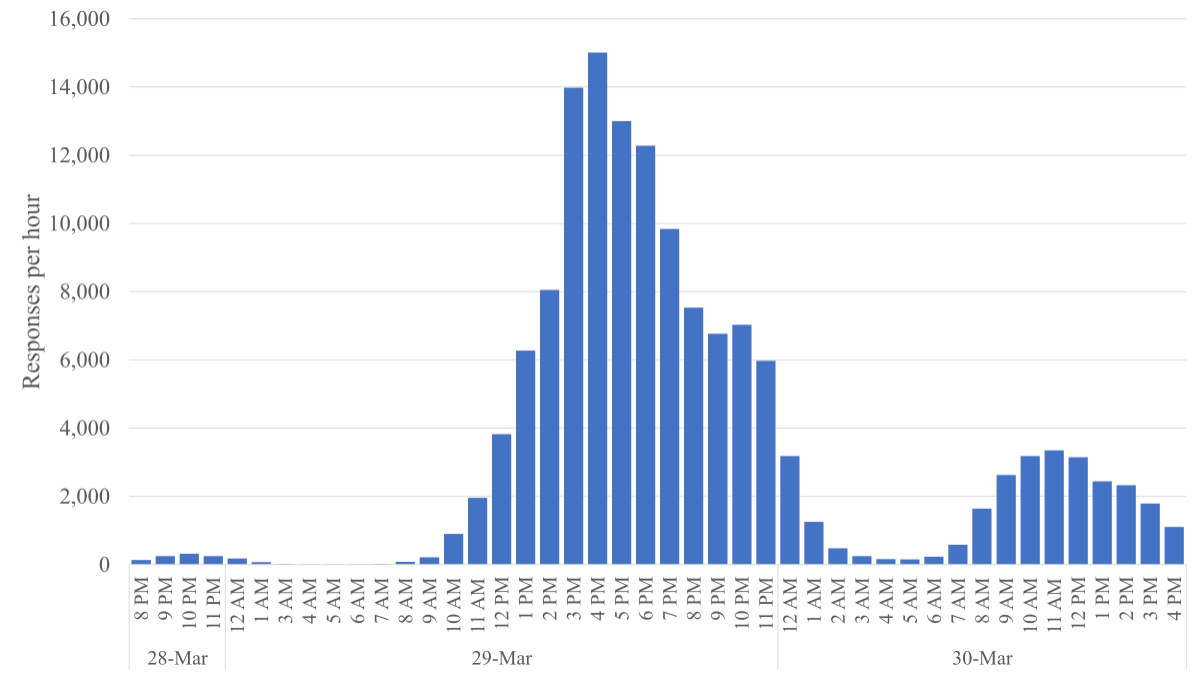
Number of answers collected by the Covid19Impact survey in its first two days, reported in one hour intervals.

Anticipating the start of new mobility restriction and social distancing measures on Monday, March 30, 2020, we deployed the survey on Saturday, March 28 at 8 PM. Via social media (Twitter and WhatsApp) and snowball sampling, we distributed the survey to a wide set of highly connected users who, in turn, distributed it to their contacts. The survey was also distributed by professional organizations, town halls, civil groups, and associations. In the 12 hours that followed, the survey went viral in Spain, and by the afternoon of Monday, March 30, we had collected over 140,000 answers. Figure 1 illustrates the growth in the number of answers over time, and the peak was reached in the time frame between 4 PM and 5 PM on Saturday, March 29, with more than 15,000 answers in 1 hour.

The initial version of the survey was delivered via Google Forms, which allowed us to write and deploy the survey in an anonymous, scalable, and free manner within hours. The URL to the Google Forms was shared via bit.ly, such that we could estimate how many times the link had been shared. After reaching 140,000 answers, we began to hit scale limitations in Google Forms, so on March 30, 2020, we moved the survey to Survey123 [26] for future editions of the data collection.

### Questionnaire Structure

All questions were anonymized to preserve privacy and no personal information was collected. In addition, the snowball sampling methodology enabled the anonymous distribution of the survey. The survey can be found online [27].

First, the survey obtained explicit consent from the users. Only when consent was granted and respondents confirmed they were adults could respondents continue to the rest of the questions.

The first section (question Q1-Q4) gathers basic demographics: country, age range, gender, and postal code. Next, there are 3 questions (Q5-Q7) related to the home situation: type of home, number of people in the home, and their ages. The following 7 questions (Q8-Q14) address the social contact behavior of the respondents during the last 2 weeks. This is an important section of the survey as we aim to understand the level of social interaction that people had despite the confinement and social distancing measures. The questions asked about having had contact with infected individuals, whether children were taken care of outside the home, if they had an external person coming to their house (eg, house cleaner), for what types of activities had they left their home, and what transportation means had they used. The last two questions intend to capture people’s perceptions of the confinement measures: if they thought the measures were enough to contain the pandemic and for how long they would be able to tolerate the containment situation. Personal economic impact is assessed with questions Q15 and Q16, followed by three questions (Q17-Q19) related to their workplace situation. Finally, the last 5 questions (Q20-Q24) address their health state to assess how many people might be infected by the virus, determine the ability of participants to self-isolate, and collect feedback regarding testing availability and testing results.

None of the questions, except for the consent question, were compulsory, and all the health-related questions included “I prefer not to answer” as a choice.

### Credibility and Validity

Before widely deploying the survey, we carried out a pilot study to validate its content and proper anonymization with a small sample of participants. The questions were written in Spanish and English. Once all the bugs were fixed and minor feedback about the wording of the questions was addressed, we proceeded to widely deploy the survey.

### Ethical Approval of the Research Protocol and Instruments

Before its deployment, the research protocol and instrument were reviewed and approved by the cabinet of the President of the Valencian Region of Spain. The findings of this survey have been regularly used and shared by the Valencian Government to assist their policy making during the COVID-19 pandemic [28].

### Data Exclusion, Cleansing, and Reweighting

From a total of 156,614 answers, we eliminated all answers with blank or invalid postal codes. Moreover, we only analyzed responses with nonblank answers related to age, gender, province, and profession (including those who reported not working), yielding a final data set of 141,865 answers.

Thus, we report the results of analyzing these 141,865 answers collected between 8 PM GMT of March 28 and 11:59 PM GMT on April 2, 2020. With such a large sample, this survey is one of the largest population surveys on COVID-19 and the largest in Spain published to date [7].

All questions were binary or categorical. Thus, we report the percentage of participants who selected each response. Because our gender, age, geographic location, and profession distributions were not proportional to those of the general population of Spain, we computed a weighting factor, such that the resulting sample had similar demographic, geographic, and profession distributions as those of Spain, reported by the Spanish National Institute of Statistics (INE). To reduce biases, we used the reweighted data for all statistical inferences. The user and home situation statistics presented in the next section correspond to the raw data without reweighting. However, the rest of the sections regarding the statistical analysis of questions Q8 to Q24 correspond to analyzing the reweighted data. Multimedia Appendix 2 contains both the raw answers and the reweighted values of the univariate tables for each of the questions. Multimedia Appendix 3 contains the 141,865 responses as a text file, with zip code and time stamp information removed to protect the anonymity of the participants.

### Statistical Analyses

The sampling error, after reweighting the samples, was 0.43. This small sampling error, due to the large sample, yields a narrow 95% CI of ±0.8428 for all proportions reported.

We use the Z test to compare two proportions, considering that the data comes from a survey and as such, the variance of each proportion is different to that of an infinite population test. We use a chi-square test to compare the independence between two questions [29]. Differences between answers greater than 0.85 were statistically significant with *P*<.001.

We measured the association between nominal variables using Cramér’s V for RxC tables and Pearson phi for 2x2 tables [30]. We used weighted logistic regression to compute the odds ratio for a multivariate model using a quasi-binomial distribution family [31].

## Results

### User Statistics and Home Situation (Q1-Q7)

Geographically, most respondents were from the Valencian Region (102,021/141,865, 71.9%). However, there were also many answers from other regions of Spain including 10,365 answers from Madrid and 5691 from Catalonia, as shown in Table 1. Multimedia Appendix 2 contains the univariate tables corresponding to all the questions in the survey, including information about the participants’ type of home and the number of people and ages of those living in their home.

**Table 1:** Age, gender, and geographical distribution of survey respondents (raw, unweighted data).

Sex, Autonomous community		Age ranges (years), n								Total (n=141,865)
		18-20 (n=3324)	21-29 (n=14,128)	30-39 (n=25,719)	40-49 (n=38,726)	50-59 (n=34,762)	60-69 (n=19,551)	70-79 (n=5093)	≥80 (n=562)	
**Female**										
	Valencia	1687	6455	11,433	17,002	15,109	7697	1681	193	61,257
	Madrid	75	563	1235	1739	1482	702	193	26	6015
	Andalucía	105	411	700	883	746	335	61	4	3245
	Catalonia	48	344	593	757	726	437	107	15	3027
	Rest of Spain	322	1346	2474	3157	2512	1196	238	30	11,275
	Total	2237	9119	16,435	23,538	20,575	10,367	2280	268	84,819
**Male**										
	Valencia	873	3571	6453	10,620	10,181	6793	2064	209	40,764
	Madrid	42	355	774	1237	1097	597	230	18	4350
	Andalucía	27	209	397	667	617	410	109	10	2446
	Catalonia	18	184	350	552	513	305	121	18	2061
	Rest of Spain	127	690	1310	2112	1779	1079	289	39	7425
	Total	1087	5009	9284	15,188	14,187	9184	2813	294	57,046

Given the gender, age and location biases in the raw data, we reweighted the data to match the distribution of the Spanish population according to the latest census [32], as reflected in Multimedia Appendix 2.

Almost all of the 141,865 respondents (n=141,807, 98.8%) lived in an apartment (n=93,060, 65.6%) or a single-family home (n=46,975, 33.1%). Most of the participants lived in a home with 2 (n=42,513, 30.0%), 3 (n=36,879, 26.0%), or 4 (n=38,265, 27.0%) people, which is consistent with Spain’s demography.

The rest of the reported statistics in this paper correspond to analyzing the reweighted sample to match in gender, age, province, and profession the distribution in Spain according to the latest data published by the Spanish INE.

Given that COVID-19’s fatality rates are largest for older adults [33], we analyzed the age distribution of the homes with older adults: 11.8% of respondents older than 50 years lived with an older adult (age>60 years) and 19.9% of respondents lived in homes inhabited only by older adults. Intergenerational homes are particularly important for the transmission of SARS-CoV-2 [34].

### Social Contact Behavior (Q8-Q14)

With respect to social contact behavior with individuals with a confirmed SARS-CoV-2 infection (Q8), 17.3% of respondents reported having had close contact with a person who was infected with COVID-19 (n=140,008). The most common social context was a coworker (6.2%), a household member (6.1%), or a friend or relative (5.4%). In the case of having been in close contact with a confirmed infected person who was a patient of the participant, a gender-centric analysis revealed a significant (*P*<.001) difference between male and female respondents: 60.7% of the respondents were female vs 39.3% male. This large difference is partially due to the larger percentage of women (72.5%) who work in the health care sector vs men (27.5%) in Spain [32].

When asked if an outside person regularly visited the home (Q10), we identified a significant difference (*P*<.001) between older adults (age >70 years) and younger respondents (n=141,365): 21.2% of older respondents regularly had a person coming to their home versus only 13.6% in the case of younger adults (age <60 years). This is an important finding as special measures might need to be taken to protect the 21.2% of older adults who regularly receive external people in their homes.

**Figure 2:**
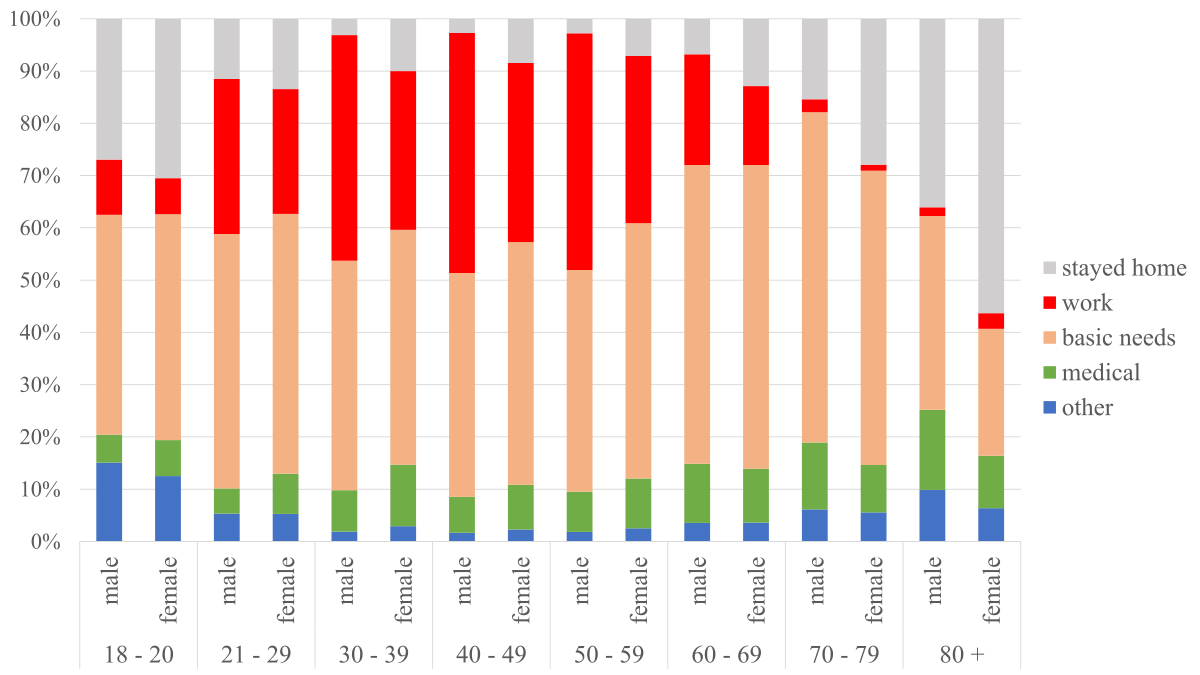
Reasons for leaving the home by gender and age.

Respondents (n=140,686) left their homes during the social distancing period for a variety of purposes (Q11), as shown in Figure 2: covering basic needs (supermarkets, bakery, and pharmacy) was the most common reason, reported by 47.8% of respondents, followed by going to work (31.3% of respondents). We identified statistically significant differences (*P*<.001) regarding age and gender. Older respondents (age >60 years) were more likely than younger participants (age <60 years) to stay entirely at home (14.9% older vs 7.6% for younger), and to leave their home to go to the pharmacy (11.5% vs 10.8%) and newspaper stand (9.7% vs 3.9%).

Conversely, younger respondents (age<60 years) were more likely to leave their home to help others than older respondents (age >60 years; 81.0% vs 71.8%). Interestingly, the youngest respondents (aged 18-29 years, n=17,416) were also more likely to stay entirely at home versus respondents 30 years or older (23.1% vs 8.2%).

Regarding gender, among all female respondents, 14.8% reported not leaving the home versus 6.5% among male respondents. This difference was statistically significant (*P*<.001). The same pattern is found with respect to leaving the home to go to work, where 26.0% of all female participants versus 36.7% of all male respondents selected this option.

The main means of transportation (Q12) used by respondents was individual (84.5%; by foot, individual car, motorcycle, scooter) versus shared (5.9%; public transport, shared car, taxi). In this question, we observed the same gender patterns as in Q11 (n=140,308): among female respondents, 13.0% reported not leaving the home versus 6.2% among male respondents.

The last two questions in this section (Q13 and Q14) concerned the personal experience of respondents regarding the containment measures; 50.4% of participants (n=141,481) believed that the government should implement more measures to contain the pandemic, and only 2.2% thought that the measures were too severe. There was a significant difference (*P*<.001) in the support of the measures by age group. Despite being at a lower risk of death, 50.0% of younger people (age <60 years) believed measures should be stronger versus 37.1% of older people (age >59 years).

Q14 (n=138,155) explored how sustainable participants consider the social distancing measures to be. The most popular answer by respondents was that they could continue in this confined state for 1 additional month (44.1%), and a nonnegligible 32.4% reported being able to continue in confinement for 3-6 months. An interesting gender difference was found for those who responded that they could stay in confinement for 6 months: among female participants, 8.0% reported this to be the case versus 12.9% among male participants (*P*<.001). This might be due to the fact that women in Spain see their workload increased during the weeks of social distancing and mobility restriction, as reported in [35].

### Personal Economic Impact and Workplace Situation (Q15-Q19)

An inevitable consequence of the COVID-19 pandemic is its economic and labor impact. Spain is a country with mostly small businesses, many of which are family owned. Q15-Q19 aim to shed light on the individual experiences and fears of people regarding their financial and employment situation.

When asked about the economic impact that the COVID-19 crisis is having on respondents’ lives (Q15, n=139,008), 43.0% felt that the crisis had not yet significantly affected them economically. Moreover, 29.1% reported that their employer or company was undergoing financial problems, and 7.7% reported having lost a significant part of their savings or their job. These results need to be taken with great caution, as the sample was collected at the end of March and early April, when the devastating economic impact of the pandemic was not yet evident.

Among the respondents who had worked in the last month, there were significant differences in the distribution of work activities, as shown in Table 2. The most affected professions included hospitality and construction. The least affected were education and public administration.

**Table 2:** Distribution of jobs between respondents who had or were in danger of losing their job/business vs those who were not (Cramér’s V=0.252).

Job categories^a^	Lost job or business, %^b^	Not lost job or business, %^b^
Administrative services	6.0	7.8
Retail	9.3	5.3
Communications	1.2	1.8
Construction	18.7	7.3
Domestic services	0.2	1.5
Education	3.1	14.2
Entertainment/arts	0.8	0.7
Essential services	2.2	9.7
Finance	1.3	4.8
Food production	3.2	3.8
Health and social services	1.8	2.8
Hospitality	29.3	13.8
Manufacturing	6.2	4.2
Other	8.8	7.4
Professional/technical/science	1.5	2.2
Public administration	0.3	5.3
Sanitation	2.7	4.4
Transportation	3.2	2.8

^a^The job categories are defined by the Spanish labor department (for the survey we only included categories with more than 1% representation in the population).

^b^Percentages are based off the weighted sample.

Small businesses have so far borne the brunt of the economic impact. For respondents (n=24,386) working in larger companies (≥100 employees), 80.1% reported that they had not yet been significantly affected versus only 42.7% of workers (n=39,052) at the smallest companies (1-9 workers) being unaffected. Among those working in small companies, 19.4% reported their companies were facing bankruptcy even at this early stage of the pandemic.

Again, there is a gender-based statistically significant difference (*P*<.001, n=139,008). In terms of having lost their jobs or savings, this option was selected by 8.3% among female participants versus 5.9% among male respondents.

With respect to the labor situation of our respondents (Q16, n=141,865), the majority (71.2%) reported working in the last month. A small fraction (5.9%) of respondents were students.

Q17 (n=98,740) focused on whether respondents had gone to work in the last week. The answers were split between the three available options: 38.3% did not go to work, 28.7% tele-worked, and 33.0% went to work.

Statistically significant gender differences (*P*<.001, n=98,740) were observed regarding working participants who did not go to work (42.0% among female participants vs 34.9% among male participants) and those who did go to work (29.1% among female participants vs 36.6% among male participants). No significant gender difference was found for those who tele-worked (28.9% among female participants vs 28.5% among male participants). In sum, female workers were significantly more likely to stay home than male workers.

Moreover, we found that the economic impact was a key factor in determining how much longer participants believed that they could continue in confinement. To explore the relationship between economic impact, age, and the willingness to stay in confinement, we built a multivariate weighted logistic regression model with *willingness and ability to stay in confinement* as a dependent variable (answers from Q14, divided into two values: 0, corresponding to answering that “at most I could continue in confinement for one week,” and 1, corresponding to answering that “I could continue in confinement for longer than one week”). As covariate variables, we used sex, age, and the answers to question Q15 (economic impact). The logistic regression model revealed a clear impact of severe economic damage on willingness to stay in confinement; those who reported not having enough money to buy food had on average more than twice the probability of reporting not willing to continue in confinement for longer than 1 week (OR 2.23, 95% CI 1.81-2.77), and those who report being unable to pay their mortgage were on average 1.54 times more likely to also report not willing to continue in confinement for longer than 1 week (OR 1.54, 95% CI 1.29-1.83). Age also mattered; according to the model, respondents younger than 21 years had on average over twice the probability to report not willing to continue in confinement for longer than 2 weeks than those 21 years and older (OR 2.06, 95% CI 1.73-2.45).

### Health State (Q20-Q24)

In the last section, Q20-Q24 asked respondents about their health. Regarding risk factors (Q20, n=135,583), we obtained a similar split between those who reported having at least one risk factor (48.3%) versus none of the listed risk factors (46.9%). In addition, 4.9% of respondents were health care workers. The risk factors that we asked participants about were hypertension, diabetes, cardiovascular disease, respiratory illness, immunosuppression, cancer, smoker (former), smoker (current), pregnancy, and health care worker.

Q21 (n=141,313) aimed to evaluate the ability of respondents to isolate themselves were they to be diagnosed with COVID-19. This is an important question given the relevance of implementing effective quarantine measures. Whereas 72.3% of respondents reported having the ability to properly isolate themselves, a nonnegligible 27.7% of respondents acknowledged not having the necessary resources to implement a proper quarantine.

In terms of age, 34.9% of respondents younger than 50 years reported not having the appropriate quarantine resources versus 21.0% of those older than 50 years. This might be due to the presence of other adults or children in the home. Indeed, 96.7% of respondents living alone (n=13,820) reported being able to self-isolate versus 68.6% of those living with other people (n=127,493), and only 5.7% of respondents younger than 50 years reported living alone when compared to 18.3% of adults 50 years and older. Moreover, when we looked at the impact of having children in the home, we observed that 41.1% of adults with children in the home (n=28,139) responded not being able to properly isolate versus 28.0% of adults without children in the home (n=67,659, *P*<.001). Among those living with older adults (n=15,124), 10.8% reported not having appropriate quarantine infrastructure at home.

To shed light on the percentage of the population that might currently be infected by SARS-CoV-2, Q22 asked respondents if they currently had any of the following symptoms that were unusual for them: difficulty breathing, dry cough, fever, headache, productive cough, anosmia, muscle pain, and sore throat; 16.8% of respondents (n=136,386) responded having at least one of the symptoms. Regarding gender, a larger percentage of women (19.0%) versus men (14.5%) reported having symptoms. This difference is statistically significant (*P*<.001). The age group who most reported having symptoms was the 30-39 years age group (n=24,839, 20.9%).

Finally, when asked whether respondents had been tested for COVID-19 (n=138,023), 87.4% felt they did not need to be tested; 6.1% were told by their doctor they should be tested, but no tests were available; 0.7% had tested negative; 0.3% had tested positive; and 0.2% were waiting for their outcomes, resulting in an overall test rate of 1.2%. We found statistically significant (*P*<.001) differences between those who exhibited any of the three symptoms (difficulty breathing, dry cough, and fever) and those who did not, and their answers regarding testing: 93.1% of those who did not have symptoms considered testing not necessary versus only 58.1% for those who had such symptoms. Table 3 depicts the responses from these two groups.

**Table 3:** Testing needs, depending on the presence of symptoms.^a^

Testing	Difficulty breathing, dry cough, or fever, %^b^	Other or no symptoms, %^b^
Negative	2.3	0.6
No need	58.1	93.1
No test available	32.5	4.9
Positive	3.9	0.2
Waiting for results	1.2	0.1
No, but need one due to being caretaker of person at risk	2.1	1.1

^a^All differences between the symptoms/no symptoms groups were statistically significant (*P*<.001), and Cramér’s V=0.327.

^b^Percentages are based off the weighted sample.

When looking at Q8 (whether respondents had close contact with an infected individual) together with Q23 (whether they had been tested for coronavirus and the results of the test), we identified an interesting pattern. Among those who had tested positive and answered Q8 (n=414), 80.9% had close contact with a known infected individual; of these, 32.4% had been through a member of the household, friend, or relative, 26.6% through a patient (health care workers), 11.1% at work, and only 1.7% through a client. Thus, over 80% of respondents with COVID-19 knew their likely source of infection. This finding is partly explained by the fact that the survey was answered during a period of confinement with reduced mobility and social contact.

Finally, we observed in the data a nonlinear relationship between testing positive and age, gender, and the ability to self-isolate (Q21). Thus, we carried out a multivariate weighted logistic regression analysis to study the relationship between these variables and found a three-way interaction between them. Females 70 years or older who reported not being able to properly isolate had on average almost twice the probability of testing positive than otherwise (OR 1.91, 95% CI 1.18-3.073).

### Prevalence

One of the goals of the survey was to make a rapid estimate of the COVID-19 prevalence in the Spanish population. The rapid rise in deaths from mid-March onward made it clear that there were far more infections than what the official case numbers reported. Model-based prevalence estimates, such as the report from Imperial College on March 30, 2020, estimated that 15% of the population had been infected to that date in Spain [21]. Large-scale, online surveys have been shown to be a useful tool to quickly estimate the percentage of currently infected individuals in the population and identify risk factors to help design measures to contain the epidemic [8,13,14]. The goal is not to replace the golden standard of seroprevalence surveys but to assess the value of a cheap and fast large-scale online survey to infer COVID-19 prevalence at a time when there was data scarcity and limited testing capacity.

Q24 asked respondents about their coronavirus test result, which provided the ground truth for building a model to predict prevalence. Among respondents reporting confirmed test results, 426 of 1345 reported testing positive, which after reweighing would translate to 235,000 positive tests in Spain’s population of 47 million. The official number of reported positive cases on April 2, 2020, was 181,859. This difference of 30% could be due to several factors, including selection bias, underreporting, or delays in the release of official test statistics [36-38].

Since at the time it was not clear which symptoms or other factors were most indicative of COVID-19 (anosmia was reported as an important symptom by March 20 [39]), we created a generalized linear model to infer the likelihood of testing positive for SARS-CoV-2 from the survey answers. In addition to symptoms, we included as independent variables gender, age, and the presence of a person with a positive coronavirus test result in the home. Our target variable was given by Q24; we selected those answers corresponding to participants who reported having tested positive (coded as 1) and those who reported having tested negative (coded as 0) for coronavirus (n=1345).

Moreover, we performed feature selection to select the features that yielded the best performing model. The selected independent variables are depicted in Table 4: a subset of the reported symptoms (Q22), whether the household already had an infected member (Q8), gender, and whether the participant’s age was older than 70 years.

**Table 4:** Selected variables and coefficients of the generalized linear model.

Variable	Estimate	SE	T value	*P* value
(Intercept)	0.12510	0.01636	7.647	<.001
Member of home infected	0.28555	0.03137	9.103	<.001
Fever	0.18569	0.03724	4.986	<.001
Dry cough	0.05834	0.02808	2.078	.04
Productive cough	–0.09785	0.03676	–2.662	.008
Muscle pain	0.07208	0.03603	2.000	.046
Loss of sense of smell	0.45410	0.03409	13.319	<.001
Age >70 years	0.17487	0.06785	2.577	.01
Male	0.08038	0.02306	3.485	<.001

**Figure 3:**
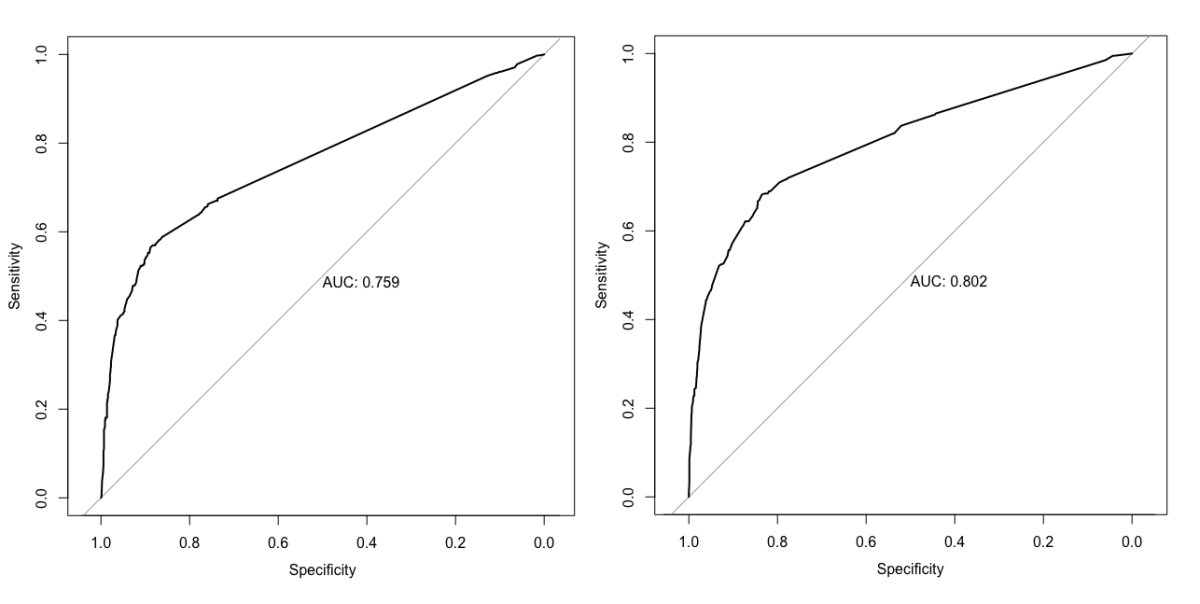
Receiver operating characteristic of the symptom-only model (left) and full model (symptom, sex, age >70 years, and household infected member model; right). AUC: area under the curve.

The obtained values from the generalized linear model were converted into probabilities by means of the logistic function (exp(x)/(1+exp(x)). The variables and parameters of the model are shown in Table 4. The final model had a sensitivity of 0.77 and a specificity of 0.80. Figure 3 (right) shows the receiver operating characteristic of this model. Although we experimented with more sophisticated machine learning models, for the purpose of this paper, we wanted to show that we could arrive at a reasonable estimate using a simple and easily reproducible method. A similar approach has been described in [6] for US and UK data.

Based on the model with the coefficients below, we estimated the number of SARS-CoV-2 positive individuals among all the respondents to the survey. Geographically, we aggregated the results by the 17 autonomous communities in Spain, making it easier to compare with official data, since each autonomous community has its own health care system, and official figures are always reported by autonomous community.

According to this model, 5798 of the respondents had likely SARS-CoV-2 infections (of which 40% were asymptomatic), which would lead to a prevalence of 5.0% (95% CI ±1.1; after rebalancing by region), suggesting that official tests were only identifying 10% of the infected individuals.

On May 13, 2020, more than a month after we had carried out our analysis, the Spanish INE published the initial results of a nationwide seroprevalence study performed between April 27 and May 11 [22,23]. This study provides ground truth data to assess the estimates of our study, both for the countrywide estimate and at a regional level.

**Figure 4:**
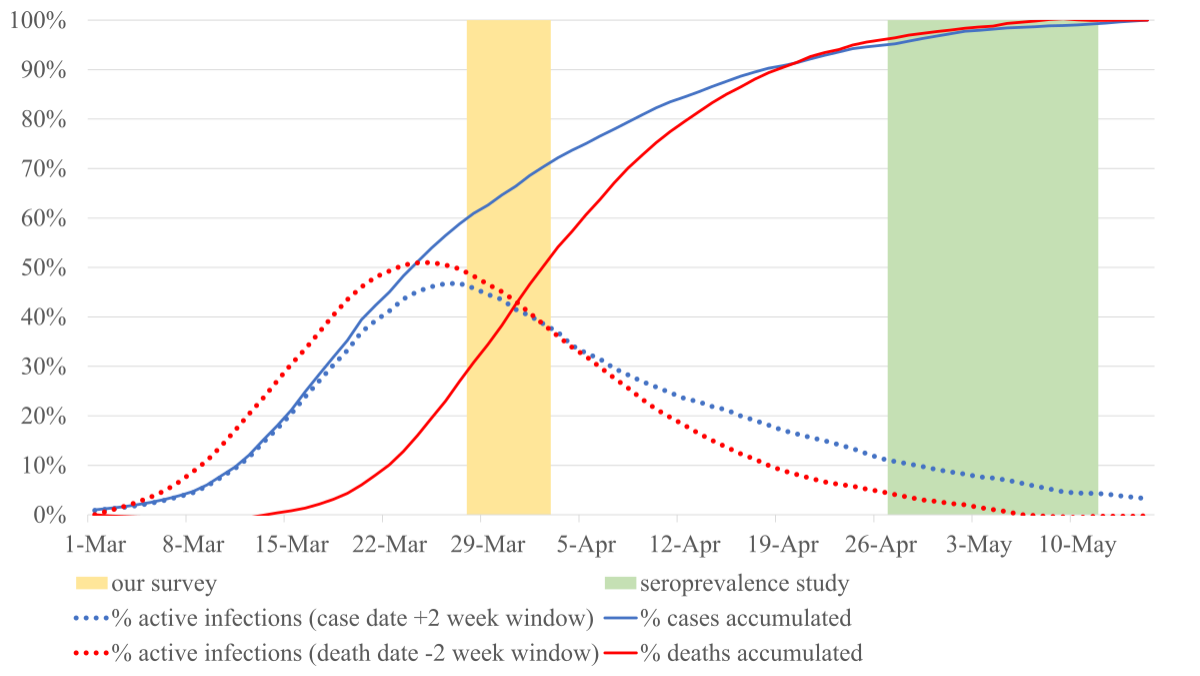
Two methods for estimating the proportion of active coronavirus infections during the time of our study in relation to those identified by the seroprevalence study. Red dotted line based on the Mortality Monitoring System deaths, assuming infection started 2 weeks prior to death. Blue dotted line based on reported positive cases, assuming infection ended 2 weeks after the case was reported. Cases and deaths from the Carlos III Health Institute in Spain.

To be able to compare our prevalence estimates with the seroprevalence study, we needed to estimate the proportion of infected individuals at the time of our survey in relation to those detected by the seroprevalence study, since the latter would capture the infected to date and, thus, would include many more individuals than those infected at the time of our survey. We performed this estimation using two different approaches, illustrated in Figure 4.

In our first estimate of the proportion, we used the excess mortality estimates provided by the Spanish Mortality Monitoring System known as MoMo [40] and made two assumptions: the number of deaths was proportional to the overall number of infected individuals and all infected individuals would have been infected for at least 2 weeks prior to death [41].

Our second estimate used the number of officially reported positive cases and assumed that individuals were infected for at least 2 weeks after their diagnosis.

As shown in Figure 4, both estimates gave similar results; according to the former, 47% (red dotted line) and, according to the latter, 45% (blue dotted line) of individuals that were detected by the seroprevalence study would have had an active SARS-CoV-2 infection during the time of our study. Note that our survey responses have a significant skew toward the beginning of our study, as 73% of the responses of our survey were collected between March 28 and 29, 2020.

In addition, we created a second model based only on symptoms. Although it had a lower area under the curve (see Figure 3, left), this model had a better defined time frame, since it only captured the people who had symptoms during the time of our study—as opposed to also capturing as-of-yet uninfected members of the household who might be infected in the future. Thus, the proportions computed previously would apply better to the symptom-only model.

Table 5 shows the prevalence estimations of each of the models based on our survey answers (symptom-only model and full model) and of the seroprevalence study for each of the 17 autonomous communities in Spain. The symptom-only model estimated a prevalence 40% lower than that of the seroprevalence study.

**Table 5:** Comparison of inferred prevalence by two models based on the survey answers and the seroprevalence study.

Autonomous community	Participants, n	Symptom-only model, % (95% CI)	Full model, % (95% CI)	Seroprevalence survey, % (95% CI)
Andalucía	5691	2.2 (±0.3)	4.4 (±0.5)	2.7 (2.2-3.2)
Aragón	1463	2.0 (±0.3)	3.1 (±0.9)	4.9 (3.8-6.3)
Asturias	655	1.5 (±0.3)	4.0 (±1.5)	1.8 (1.3-2.5)
Balearic Islands	1222	1.9 (±0.3)	4.2 (±1.1)	2.4 (1.6-3.5)
Canarias	1052	1.4 (±0.2)	3.0 (±1.0)	1.8 (1.1-2.8)
Cantabria	497	2.8 (±0.3)	4.6 (±1.8)	3.2 (2.1-5.0)
Castilla y León	1994	3.7 (±0.4)	6.1 (±1.0)	7.2 (6.3-8.1)
Castilla-La Mancha	3469	8.0 (±0.3)	10.4 (±1.0)	10.8 (9.3-12.4)
Catalonia	5088	2.8 (±0.3)	4.8 (±0.6)	5.9 (4.9-6.9)
Valencia	102,021	1.6 (±0.3)	3.4 (±0.1)	2.5 (1.9-3.2)
Extremadura	656	2.3 (±0.4)	4.4 (±1.6)	3.0 (2.2-4.1)
Galicia	2257	1.3 (±0.3)	2.6 (±0.7)	2.1 (1.7-2.6)
Madrid	10,365	6.1 (±0.4)	8.8 (±0.5)	11.3 (9.8-13.0)
Murcia	3566	1.5 (±0.3)	3.2 (±0.6)	1.4 (0.8-2.4)
Navarra	580	3.6 (±0.4)	5.5 (±1.9)	5.8 (4.3-7.6)
País Vasco	1007	1.9 (±0.4)	3.9 (±1.2)	4.0 (3.1-5.2)
Rioja, La	220	1.8 (±0.4)	5.0 (±2.9)	3.3 (2.4-4.4)
National	141,803	3.0 (±0.3)	5.0 (±1.1)	5.0 (4.7-5.4)

The prevalence estimates by the full model are closer to the estimates provided by the seroprevalence study. This finding might be explained by the fact that 40% of the identified likely infections by the full model were not based on symptoms, but instead based on the variable that captures if the respondents shared their home with an infected individual. Due to the harsh nature of the lockdown in Spain at that time, including the banning of all mobility except essential labor and basic needs between March 30 and April 9, 2020 (both included) [42], many of the new infections in the period between the end of our study and the end of the seroprevalence survey (ie, between April 4 and May 17) may have been household members captured by our model.

## Discussion

### Principal Findings

Through the survey answers, we identified several patterns and implications for the design of public policies in the context of the COVID-19 pandemic.

First, our work highlights the value of involving the population and carrying out large-scale online surveys for a quick assessment of the situation and perceptions during a pandemic. We were overwhelmed by the response to the survey. Mayors in large and small towns got involved and shared it with their employees and residents, professional and civic associations disseminated it among their members, individuals advertised it among their contacts, and a few media organizations gave it visibility via articles and posts. This outstanding response by people might reflect a societal need to have more information about the impact of the COVID-19 pandemic in our lives and is an example of citizen’s science and people’s willingness to help by contributing with their answers to achieve more data-driven decision-making processes. Although the sample has some biases, we used reweighting to mitigate them.

Second, we empirically corroborate the impact that close contacts play in the transmission of the disease. Over 16% of respondents reported having had close contact with someone who was infected by SARS-CoV-2. This percentage was much higher (80.9%) among those who had tested positive for coronavirus. According to this finding, those testing positive were likely infected by someone they knew and had close contact with, rather than, for example, an unknown infected stranger in a supermarket. This finding could have implications for contact tracing strategies.

Third, gender matters. Several statistically significant differences were found between male and female respondents, with a pattern of placing women in situations of higher vulnerability or exposure when compared to men. As in other aspects of society, gender-based differences exist in the context of a pandemic. It is a socially important factor that needs to be considered.

Fourth, age also matters. We identified statistically significant differences in the social contact behavior questions between older participants (age >60 years) and younger participants (age <60 years). Older respondents were almost twice as likely to stay entirely at home than younger participants. There were also different aged-based attitudes toward the containment measures; younger participants were significantly more supportive of stronger measures than older participants, while they were more likely to report not being able to stand the confinement any more (4.9%) versus older adults (0.8%). We also found associations between age, gender, the ability to self-isolate, and the probability of testing positive; older females without the capacity to isolate themselves were almost twice as likely to test positive than otherwise.

Participants demanded more measures, as 50.4% of respondents were supportive of implementing additional social distancing measures. This result might reflect the worry in people’s minds regarding the exponential progression of the pandemic and the lack of clear signs of flattening the curve at the time of answering the survey.

Moreover, the majority of respondents (76.5%) were willing to remain in confinement for a month or more, and 32.4% of respondents reported being able to do so for 3-6 additional months.

Even at the end of March, the economic impact of the pandemic was evident, particularly for those working in small companies, 19.4% of which reported to be facing bankruptcy. Moreover, over 47.3% of participants who worked in small companies reported having been impacted by the pandemic. In terms of professions, hospitality, construction, and retail were the most affected. Hospitality represents 6.2% of the Spanish GDP [43] and construction 5.6% [44]. We expect the economic impact to be significantly larger as the pandemic progresses.

Among those who were working, 28.7% of respondents reported tele-working and one-third leaving the home to go to work. The tele-work figure is lower than in other countries. For example, in the United States, it is estimated that 56%-62% of the workforce could work remotely. Moreover, on March 31, 2020, the government established labor mobility restrictions for all nonessential professions. Given that 71.2% of respondents (n=141,865) reported having worked in the last month, our expectation is that about 23% of the population would have been impacted by such measures. Regarding workplace infections, we found that 11.1% of those who tested positive (and did not work in the health care sector) had close contact with someone at work who had tested positive for coronavirus.

Close, known contacts seem to play a large role on infections, as 80.9% of those who had tested positive responded having had close contact with a known infected individual. This finding is relevant in the design of contact tracing, testing, and isolation strategies.

Quarantine infrastructure might be needed, as over 27.7% of respondents reported not having the appropriate infrastructure to isolate themselves at home. Effective quarantine measures for asymptomatic or lightly symptomatic patients are key to control the spread of the pandemic. Thus, developing the needed infrastructure might be key to slowdown the transmission of the disease.

The number of SARS-CoV-2 infected individuals in Spain in March and April was certainly larger than what has been officially reported. In our survey, over 16.8% of respondents reported having at least one possibly COVID-19–related symptom. We show how the prevalence of a rapidly spreading disease such as COVID-19 can be estimated using a large-scale population survey. From the answers to two of the questions (Q22: symptoms and Q8: contact with coronavirus-infected individual in the household) plus demographic information, we built a generalized linear model and reported SARS-CoV-2 prevalence estimations that are on par with those carried out by a seroprevalence study in Spain. As shown in related studies [6], when public policy decisions need to be made rapidly in a situation of data scarcity and limited testing capacity, a large-scale population survey might be of great value to make fast assessments of prevalence, as it can be deployed rapidly and enable the collection of results within hours.

Finally, in the context of Spain, our survey revealed a lack of tests; over 6.1% of respondents reported not being able to do the test despite their doctor’s recommendation. Moreover, a significant difference was found between those who had at least one of three COVID-19 symptoms, namely, dry cough, fever, and difficulty breathing, and those who did not regarding the impact of testing unavailability; 32.5% of the symptomatic individuals reported that tests were not available despite their doctor’s recommendation. Thus, we found that there was a need for more tests.

### Limitations

Although the sample size in our study is large, our methodology is not exempt of limitations. First, there are several sources of bias in our study: selection bias, given that all participants volunteered to fill out the survey without any incentive; self-reported bias; and sampling bias as we used a nonprobability sampling technique. Geographically, we lacked representation of several geographic regions in Spain and particularly rural areas. In our analysis, we tried to mitigate some of these biases by correcting for gender, age, location, and profession via reweighting using the Spanish INE census data. However, reweighting does not eliminate the risk of selection bias. Second, this is an in-the-wild study, and thus, people could have provided untruthful answers. We addressed this limitation by filtering entries without proper zip codes and entries that had inconsistencies in them. In addition, our study provides a snapshot over a 5-day time period, such that the results are only representative of this time period. We have addressed this limitation by deploying the study on a weekly basis since its first deployment at the end of March [28].

Finally, our prevalence estimates have several limitations, including overestimation of symptoms due to the existence of other flu-like illnesses at the time, and selection bias for symptomatic individuals who might have been more motivated to respond or forward the survey. We also lacked detail regarding which SARS-CoV-2 test respondents had taken, the reliability of these tests, and the relative timeframe of when the tests were taken versus symptoms. However, as noted in the manuscript, our goal is to show the value of large-scale, online, self-reported population studies to quickly and cheaply approximate the prevalence of COVID-19 when testing capacity is limited and data is scarce, as was the case in Spain at the time of the study and as might be the case in other countries in the future.

### Conclusions

The COVID-19 pandemic is undoubtedly having a major impact on the lives of people worldwide. Although there is data regarding the number of reported cases, hospitalizations and intensive care patients, and deaths, there is a scarcity of data about the individual experiences of people; their personal, financial and labor situations; their health state; and their attitudes toward the confinement measures. This paper reports the first results of analyzing a large-scale, rich data set of self-reported information regarding the social contact, economic impact, working situation, and health status of over 140,000 individuals in Spain. It is the largest population survey carried out in Spain in the context of an infectious disease pandemic.

The data is extremely rich and multifaceted. Thus, it offers numerous avenues of future work and deeper analysis according to different dimensions, including location (at a zip code level), which we have not covered in this paper.

We have launched successive versions of the *Covid19Impact* survey [27] in consecutive weeks throughout the COVID-19 pandemic to assess the COVID-19 situation from the perspective of the population in Spain over time and identify changes in people’s situations and perceptions regarding the pandemic.

## Appendix

Multimedia Appendix 1 Survey questions translated from Spanish.

Multimedia Appendix 2 Univariate tables.

Multimedia Appendix 3 A .txt file is provided with the 141,865 survey answers used in this paper. To protect the anonymity of the survey respondents, only province-level geographic location information is included, the timestamp has been removed, and the answers have been randomly sorted to prevent deanonymization.

## Abbreviations

GDP: gross domestic product
INE: National Institute of Statistics
Q: question
