# Supplementary material for: Assessing the Impact of the COVID-19 Pandemic in Spain: Large-Scale, Online, Self-Reported Population Survey
Source: J Med Internet Res. 2020 Sep 10;22(9):e21319. doi: 10.2196/21319 (PMC7485997; doi:10.2196/21319)
Supplement: Multimedia Appendix 1 [file jmir_v22i9e21319_app1.docx]

**Appendix 1. Survey Questions**
*(Translated from Spanish)* [*http://covid19impactsurvey.org*](http://covid19impactsurvey.org)

| Start | Consent | I am an adult and I consent to taking this survey  I am not an adult, or I do not consent taking this survey *(skip to end)* |
| --- | --- | --- |
|  | Q1 In which country are you presently in? | Spain and other Latin American countries |
| Basic data | Q2 What is your age range? | 18-20  21-29  30-39  40-49  50-59  60-69  70-79  80 or more |
|  | Q3 What is your gender? | Male  Female |
|  | Q4 Postal code | Entered as text |
| Home situation | Q5 Type of home | Single Family  Apartment  Old age home  Home for disabled people  Prison/Jail  Hotel  Other shared accommodation (monastery, etc.)  Camping  Homeless  Other |
|  | Q6 Number of people in home (including you) | 1  2  3  4  5 or more |
|  | Q7 Age(s) of people in your home  *(check all that apply)* | 10 or less  11-20  21-29  30-39  40-49  50-59  60-69  70-79  80 or more |
| Social contact in the last two weeks | Q8 Have you had physical contact with someone diagnosed with coronavirus? *(check all that apply)* | None that I know of  Member of household  Family outside household  Friend  Coworker  Cleaning staff/nurse/etc.  Patient (in case of medical staff)  Client/Customer |
|  | Q9 If you have children, are they taken care of by someone outside the home (grandparents, neighbors, etc.)? | Yes  No  I don't have children |
|  | Q10 Does anyone who doesn't live in your home regularly enter (cleaner, nurse, caretaker, etc.)? | Yes  No |
|  | Q11 For what activities do you leave your home? *(check all that apply)* | Go to hospital  Go to a doctor's appointment  Go to a health care center (blood test, anticoagulants, etc.)  Go to work  Go to supermarket  help someone that lives outside your home  Go to the bank  Go to the pharmacy  Go to the bakery  Go to the newspaper stand  Walk the dog  Other  Stayed home the whole time |
|  | Q12 What means of transport do you use? *(check all that apply)* | Walk  Motorcycle  Car (individual)  Car (shared)  Bike/scooter  Public transport (bus, train. etc.)  Taxi/Uber/etc.  Stayed home |
|  | Q13 Do you believe that the measures the government have taken are enough to contain the spread of coronavirus? | No, should be stricter  Yes, are about right  Yes, but are too strict  Prefer not to respond  Don't know |
|  | Q14 If you are currently confined to not leaving your home, how much longer can you stand it? | 0 days, I can't stand it anymore  1 week  2 weeks  1 month  2 months  6 months |
| Economic impact | Q15 What kind of economic impact has the coronavirus had on you? (check all that apply) | No or little impact  I lost my job  I lost my savings  I can't pay my mortgage anymore  I can't afford to buy food  My business is in danger of bankruptcy |
|  | Q16 Have you gone to work in the last month? | Yes  No  No, I'm a student |
| Workplace *(skip unless the previous answer was yes)* | Q17 Have you gone to work in the last week? | Yes  No  No, but I'm teleworking |
|  | Q18 How many people work at your place of work? | 1-9  10-99  100+ |
|  | Q19 What is your main type of work? | Essential services (police, fireman, doctor)  Retail large/small  Manufacturing  Health and social services  Hospitality  Education  Government or defense  Construction  Transport  Administrative assistant and similar  Professional, technical, scientist  Farming, fishing or other food production  Press or communication  Domestic care  Financial  Arts, entertainment, recreation  Sanitation, cleaning, garbage collection  Other services |
| Health | Q20 Are you a member of any of these risk groups? *(check all that apply)* | Hypertension  Diabetes  Cardiovascular disease  Respiratory illness  Immuno-suppressant  Cancer  Smoker (current)  Smoker (ex)  Pregnant  Health care worker  Not in a risk group  I prefer not to answer |
|  | Q21 If you were diagnosed with coronavirus, would you be able to isolate yourself from other members in your home? | Yes  No |
|  | Q22 Do you have any of the following symptoms (more than normal) *(check all that apply)* | Fever  Dry cough  Productive cough  Difficulty breathing  Sore throat  Headache  Muscle pain  Loss of sense of smell  None of these symptoms  I prefer not to answer |
|  | Q23 How long have you had these symptoms? | I don’t have these symptoms  1 - 3 days  4 - 7 days  8 - 13 days  14 or more days  I prefer not to answer |
|  | Q24 Have you taken the test for coronavirus? | No, but I don't think I need it  No, my doctor recommended it but there weren't any tests available  Yes, I'm waiting for my result  Yes, the result is I have COVID-19  Yes, the result is I don't have COVID-19  I prefer not to answer |
