# Supplementary material for: Assessing the Impact of the COVID-19 Pandemic in Spain: Large-Scale, Online, Self-Reported Population Survey
Source: J Med Internet Res. 2020 Sep 10;22(9):e21319. doi: 10.2196/21319 (PMC7485997; doi:10.2196/21319)
Supplement: Multimedia Appendix 2 [file jmir_v22i9e21319_app2.docx]

# Univariate tables

# Q2 What is your age range?

| **Q2** | **N** | **%** | **% weighted** | **INE^+^ %** |
| --- | --- | --- | --- | --- |
| **<30** | 17,452 | 12.3 | 13.6 | 13.9 |
| **30-39** | 25,719 | 18.1 | 18 | 18.1 |
| **40-49** | 38,726 | 27.3 | 22.5 | 22.3 |
| **50-59** | 34,762 | 24.5 | 19.8 | 19.7 |
| **60-69** | 19,551 | 13.8 | 14.9 | 14.8 |
| **70-79** | 5,093 | 3.6 | 10.8 | 10.8 |
| **80+** | 562 | 0.4 | 0.3 | 0.3 |
| **Total** | 141,865 |  |  |  |

INE: Spanish National Institute of Statistics

# Q3 What is your gender?

| **Q4** | **N** | **%** | **% weighted** | **INE^+^ %** |
| --- | --- | --- | --- | --- |
| **Female** | 84,819 | 59.8 | 50.8 | 50.9% |
| **Male** | 57,046 | 40.2 | 49.2 | 49.1% |
| **Total** | 141,865 |  |  |  |

# Q5 Type of home

| **Q5** | **N** | **%** | **% weighted** |
| --- | --- | --- | --- |
| **appartment** | 93,060 | 65.62 | 66.28 |
| **camping** | 45 | 0.03 | 0.04 |
| **disabled_home** | 10 | 0.01 | 0.01 |
| **homeless** | 29 | 0.02 | 0.02 |
| **hotel** | 31 | 0.02 | 0.04 |
| **old_age_home** | 36 | 0.03 | 0.03 |
| **other** | 1,338 | 0.94 | 0.86 |
| **other_shared** | 255 | 0.18 | 0.23 |
| **prison** | 28 | 0.02 | 0.04 |
| **single_family** | 46,975 | 33.13 | 32.46 |
| **Total** | 141,807 |  |  |

# Q6 Number of people in your home (including yourself)

| **Q6** | **N** | **%** | **% weighted** |
| --- | --- | --- | --- |
| **1** | 13,969 | 9.8 | 11.1 |
| **2** | 42,513 | 30 | 31.6 |
| **3** | 36,879 | 26 | 24.2 |
| **4** | 38,265 | 27 | 25.1 |
| **5+** | 10,239 | 7.2 | 8 |
| **Total** | 141,865 |  |  |

# Q7* Ages of people in your home (check all that apply)

| **Q7** | **N** | **%** | **% weighted** |
| --- | --- | --- | --- |
| **<10** | 32,666 | 23.7 | 20.6 |
| **11-20** | 35,646 | 25.9 | 25.2 |
| **21-29** | 28,742 | 20.9 | 19.5 |
| **30-39** | 30,030 | 21.8 | 21.7 |
| **40-49** | 42,766 | 31.1 | 28.9 |
| **50-59** | 44,396 | 32.2 | 30.3 |
| **60-69** | 26,257 | 19.1 | 19.8 |
| **70-79** | 8,934 | 6.5 | 11.9 |
| **80+** | 5,362 | 3.9 | 3.7 |
| **Total** | 137,704 |  |  |

* Multiple answer question

# Q8* Have you had physical contact with someone diagnosed with coronavirus?

| **Q8** | **N** | **%** | **% weighted** |
| --- | --- | --- | --- |
| **no_one** | 119,095 | 85.1 | 82.8 |
| **home_member** | 7,172 | 5.1 | 6.1 |
| **family** | 3,443 | 2.5 | 2.8 |
| **friend** | 2,487 | 1.8 | 2.6 |
| **coworker** | 7,577 | 5.4 | 6.2 |
| **cleaning_person** | 683 | 0.5 | 0.7 |
| **sick_patient** | 3,443 | 2.5 | 2.6 |
| **professional_client** | 1,219 | 0.9 | 1 |
| **Total** | 140,008 |  |  |

# Q9 If you have children, are they taken care of by someone outside the home (grandparents, neighbors, etc…)?

| **Q9** | **N** | **%** | **% weighted** |
| --- | --- | --- | --- |
| **No** | 82,479 | 59.2 | 56.7 |
| **No kids** | 48,341 | 34.7 | 37.4 |
| **Yes** | 8,535 | 6.1 | 5.9 |
| **Total** | 139,355 |  |  |

# Q10 Does anyone who does not live in your home regularly enter your house (e.g. cleaner, nurse…)?

| **Q10** | **N** | **%** | **% weighted** |
| --- | --- | --- | --- |
| **No** | 121,657 | 86.1 | 85.3 |
| **Yes** | 19,708 | 13.9 | 14.7 |
| **Total** | 141,365 |  |  |

# Q11* For what activities do you leave your home? (check all that apply)

| **Q11** | **N** | **%** | **% weighted** |
| --- | --- | --- | --- |
| **Hospital** | 7,265 | 5.1 | 5.3 |
| **Doctor appointment** | 5,333 | 3.8 | 3.8 |
| **Health center** | 7,202 | 5.1 | 4.7 |
| **Work** | 44,593 | 31.4 | 32.0 |
| **Supermarket** | 112,567 | 79.3 | 79.1 |
| **Help others** | 15,836 | 80  11.2 | 9.5 |
| **Bank** | 15,241 | 10.7 | 11.0 |
| **Pharmacy** | 58,074 | 40.9 | 40.9 |
| **Bakery** | 41,142 | 24.1 | 23.1 |
| **Kiosk** | 23,058 | 21.2 | 21.2 |
| **Walk dog** | 20,440 | 14.4 | 12.8 |
| **Other** | 12,26 | 8.6 | 8.6 |
| **Stayed home** | 1179 | 0.8 | 1.0 |
| **Total** | 140,686 |  |  |

# Q12* What means of transport do you use? (check all that apply)

| **Q12** | **N** | **%** | **% weighted** |
| --- | --- | --- | --- |
| **Walk** | 78,998 | 56.1 | 55.6 |
| **Bike** | 1,144 | 0.8 | 0.8 |
| **Public transport** | 3,005 | 2.1 | 2.8 |
| **Motorcycle** | 2,110 | 1.5 | 2 |
| **Car shared** | 3,174 | 2.3 | 2.7 |
| **Car individual** | 77,751 | 55.2 | 53.1 |
| **Stayed home** | 12,511 | 8.9 | 9.6 |
| **Taxi** | 852 | 0.6 | 0.7 |
| **Total** | 140,799 |  |  |

# Q13 Do you believe that that measures the government have taken are enough to contain the spread of the coronavirus?

| **Q13** | **N** | **%** | **% weighted** |
| --- | --- | --- | --- |
| **Do more** | 65,453 | 49.4 | 50.4 |
| **Enough** | 36,624 | 27.7 | 27.3 |
| **Too much** | 2,422 | 1.8 | 2.2 |
| **Don’t know** | 27,899 | 21.1 | 20.1 |
| **Total** | 141,481 |  |  |

# Q14 If you are currently confined to not leaving your home, how much longer can you stand it?

| **Q14** | **N** | **%** | **% weighted** |
| --- | --- | --- | --- |
| **I can’t anymore** | 1,877 | 1.4 | 1.5 |
| **1 week** | 4,108 | 3 | 3.2 |
| **2 weeks** | 26,473 | 19.2 | 18.9 |
| **1 month** | 61,412 | 44.5 | 44.1 |
| **3 months** | 30,134 | 21.8 | 21.9 |
| **6 months** | 14,151 | 10.2 | 10.5 |
| **Total** | 138,155 |  |  |

# Q15* What kind of economic impact has the coronavirus had on you? (check all that apply)

| **Q15** | **N** | **%** | **% weighted** |
| --- | --- | --- | --- |
| **None** | 93,132 | 65.6 | 63 |
| **Lost job** | 9,322 | 6.6 | 8.1 |
| **Lost savings** | 10,634 | 7.5 | 7.8 |
| **Can’t pay mortgage** | 10,449 | 7.4 | 7.9 |
| **No food** | 3,644 | 2.6 | 2.6 |
| **Company bankrupt** | 11,039 | 7.8 | 9.2 |
| **Employer bankrupt** | 956 | 0.7 | 0.9 |
| **Total** | 141,865 |  |  |

# Q16 Have you gone to work in the last month?

| **Q16** | **N** | **%** | **% weighted** |
| --- | --- | --- | --- |
| **No** | 36,804 | 25.9 | 22.8 |
| **No, I’m student** | 6,640 | 4.7 | 5.9 |
| **Yes** | 98,421 | 69.4 | 71.2 |
| **Total** | 141,865 |  |  |

# Q17 Have you gone to work in the last week?

| **Q17** | **N** | **%** | **% weighted** |
| --- | --- | --- | --- |
| **No** | 32,150 | 32.6 | 38.3 |
| **Teleworking** | 32,787 | 33.2 | 28.7 |
| **Yes** | 33,803 | 34.2 | 33 |
| **Total** | 98,740 |  |  |

# Q18 How many people work at your place of work?

| **Q18** | **N** | **%** | **% weighted** |
| --- | --- | --- | --- |
| **100+** | 24,386 | 25 | 25.1 |
| **10-99** | 33,947 | 34.9 | 33.4 |
| **1-9** | 39,052 | 40.1 | 41.5 |
| **Total** | 97,385 |  |  |

# Q19 What is your main type of work?

| **Q19** | **N** | **%** | **% weighted** |
| --- | --- | --- | --- |
| **Admin services** | 5,327 | 5.4 | 7.6 |
| **Retail large/small** | 7,164 | 7.3 | 5.7 |
| **Press or communication** | 3,549 | 3.6 | 1.8 |
| **Construction** | 2,723 | 2.8 | 8.7 |
| **Domestic care** | 883 | 0.9 | 1.4 |
| **Education** | 16,879 | 17.1 | 12.9 |
| **Entertainmnet** | 2,023 | 2.1 | 0.7 |
| **Essential services (police, fireman, doctor)** | 7,692 | 7.8 | 8.9 |
| **Financial** | 3,064 | 3.1 | 4.4 |
| **Farming, fishing or other food production** | 1,633 | 1.7 | 3.8 |
| **Health and social services** | 7,425 | 7.5 | 2.7 |
| **Hospitality** | 3,525 | 3.6 | 15.7 |
| **Manufacturing** | 4,507 | 4.6 | 4.4 |
| **Other services** | 12,296 | 12.5 | 7.5 |
| **Professional, technical, scientist** | 8,475 | 8.6 | 2.1 |
| **Government or defense** | 8,607 | 8.7 | 4.7 |
| **Sanitation, cleaning, garbage collection** | 628 | 0.6 | 4.2 |
| **Transport** | 2,261 | 2.3 | 2.8 |
| **Total** | 98,661 |  |  |

# Q20* Are you a member of any of these risk groups? (check all that apply)

| **Q20** | **N** | **%** | **% weighted** |
| --- | --- | --- | --- |
| **Hypertension** | 17,387 | 12.3 | 14.4 |
| **Diabetes** | 5,133 | 3.6 | 4.2 |
| **Cardiovasular** | 4,677 | 3.3 | 4.1 |
| **Respiratory** | 8,421 | 5.9 | 6.3 |
| **Immunocompromised** | 2,926 | 2.1 | 2 |
| **Cancer** | 2,674 | 1.9 | 2.1 |
| **Smoker** | 42,429 | 29.9 | 30 |
| **Exsmoker** | 17,324 | 12.2 | 12.5 |
| **Pregnant** | 1,039 | 0.7 | 0.5 |
| **Healthcare worker** | 8,000 | 5.6 | 4.6 |
| **None** | 65,074 | 45.9 | 44.4 |
| **Prefer not to say** | 5,819 | 4.1 | 4.5 |
| **Total** | 141,865 |  |  |

# Q21 If you were diagnosed with coronavirus, would you be able to isolate yourself from other members of your home?

| **Q21** | **N** | **%** | **% weighted** |
| --- | --- | --- | --- |
| **No** | 40,083 | 28.4 | 27.7 |
| **Yes** | 101,230 | 71.6 | 72.3 |
| **Total** | 141,313 |  |  |

# Q22* Do you have any of the following symptoms (more than normal)? (check all that apply)

| **Q22** | **N** | **%** | **% weighted** |
| --- | --- | --- | --- |
| **Fever** | 2,007 | 1.4 | 1.7 |
| **Dry cough** | 6,835 | 4.8 | 5.2 |
| **Productive cough** | 6,353 | 4.5 | 4.4 |
| **Difficulty breathing** | 2,125 | 1.5 | 1.6 |
| **Sore throat** | 7,924 | 5.6 | 5.3 |
| **Headache** | 5,780 | 4.1 | 4.2 |
| **Muscle pain** | 3,629 | 2.6 | 2.8 |
| **Loss of smell** | 2,793 | 2 | 2.6 |
| **None** | 113,888 | 80.3 | 79.2 |
| **Prefer not to say** | 5,268 | 3.7 | 4.5 |
| **Total** | 141,865 |  |  |

# Q23 How long have you had these symptoms for?

| **Q23** | **N** | **%** | **% weighted** |
| --- | --- | --- | --- |
| **1-3** | 6,045 | 4.7 | 4.8 |
| **4-7** | 5,794 | 4.5 | 4.8 |
| **8-13** | 4,348 | 3.3 | 3.6 |
| **14+** | 5,085 | 3.9 | 4.1 |
| **No symptoms** | 107,792 | 83.0 | 82.2 |
| **Prefer not to say** | 262 | 0.6 | 0.5 |
| **Total** | 129,064 |  |  |

# Q24 Have you taken the test for coronavirus?

| **Q24** | **N** | **%** | **% weighted** |
| --- | --- | --- | --- |
| **Prefer not to say** | 5,164 | 3.7 | 3.6 |
| **Yes, result is I do not have COVID-19** | 919 | 0.7 | 0.7 |
| **No, my doctor recommended but there are no tests available** | 8,412 | 6.1 | 6.9 |
| **No, but I do not think I need it** | 121,323 | 87.9 | 86.9 |
| **No, but would want to as I am a caretaker** | 1,518 | 1.1 | 1.2 |
| **Yes, the result is I have COVID-19** | 426 | 0.3 | 0.5 |
| **Yes, I am waiting for my results** | 261 | 0.2 | 0.2 |
| **Total** | 138,023 |  |  |
